# A Mother's Ordeal

**Published:** 1920-01-24

**Authors:** 


THE PUBLIC WELL-BEING—(*continued*).

### A Mother's Ordeal.

Regard for the welfare of deserted, and especially of unmarried mothers, is a valuable step forward, though it has taken a considerable time for ill-conceived public opinion and official rectitude to come round to the humane view of the treatment and care of unmarried mothers. There is another point of importance in the Ministry's suggestion that adequate lying-in accommodation should be provided. The necessity for this was manifested recently in the distressing circumstances wherein a Southwark woman was taken in a taxicab to three hospitals before she was able to gain admission to undergo a confinement operation. The matter came before the Lambeth Board of Guardians, and the hospitals concerned were severely criticised. But it is easy to criticise; if a hospital has no accommodation to meet a sudden emergency of that kind, what is it to do? With a little notice it might, doubtless, contrive some plan. Even if a hospital is prepared for such emergency cases, there is always the possibility that one emergency may follow another, and emergency accommodation cannot be limitless. Perhaps some hospital co-ordination might meet the point, so that a patient might be directed immediately to a hospital that at the time is known to be capable of receiving the case.

